# Cardiovascular and Metabolic Consequences of Liver Transplantation: A Review

**DOI:** 10.3390/medicina55080489

**Published:** 2019-08-15

**Authors:** Oana Plotogea, Madalina Ilie, Vasile Sandru, Alexandru Chiotoroiu, Ovidiu Bratu, Camelia Diaconu

**Affiliations:** 1 Faculty of Medicine, University of Medicine and Pharmacy “Carol Davila”, 050474 Bucharest, Romania; 2 Gastroenterology Clinic, Clinical Emergency Hospital of Bucharest, 014461 Bucharest, Romania; 3 Surgery Clinic, Clinical Emergency Hospital of Bucharest, 014461 Bucharest, Romania; 4 Urology Clinic, Emergency University Central Military Hospital, 010242 Bucharest, Romania; 5 Internal Medicine Clinic, Clinical Emergency Hospital of Bucharest, 014461 Bucharest, Romania

**Keywords:** liver transplantation, cardiovascular complications, metabolic consequences, hypertension, dyslipidemia

## Abstract

Liver transplantation (LT) is considered the curative treatment option for selected patients who suffer from end-stage or acute liver disease or hepatic malignancy (primary). After LT, patients should be carefully monitored for complications that may appear, partially due to immunosuppressive therapy, but not entirely. Cardiovascular diseases are frequently encountered in patients with LT, being responsible for high morbidity and mortality. Patients with underlying cardiovascular and metabolic pathologies are prone to complications after the transplant, but these complications can also appear de novo, mostly associated with immunosuppressants. Metabolic syndrome, defined by obesity, hypertension, dyslipidemia, and hyperglycemia, is diagnosed among LT recipients and is aggravated after LT, influencing the long-term survival. In this review, our purpose was to summarize the current knowledge regarding cardiovascular (CV) diseases and the metabolic syndrome associated with LT and to assess their impact on short and long-term morbidity and mortality.

## 1. Introduction

Scientific literature about the history of liver transplantation (LT) [1,2,3] shows that the first human orthotopic LT was performed in 1963 by Thomas E. Starzl, on a three-year-old child with biliary atresia, who died during the operation. After four years of unsuccessful attempts, Starzl performed liver transplantation on a patient with hepatoblastoma who survived 18 months, but then died because of metastases [4]. Over the years, advances regarding technical surgical aspects, donor organ quality, perioperative management, the introduction of immunosuppressants, and the standardization of indications and contraindications have transformed experimental LT into a life-saving procedure, with a high impact on survival and quality of life [5]. According to the European Association for the Study of the Liver (EASL), survival rates are high: 96% at one year after LT and 71% at ten years [6].

There are multiple indications for LT, but patients should be carefully selected. Patients who may benefit the most are those in whom LT would prolong the survival beyond the life expectancy induced by the hepatic disease (usually less than one year) [6]. LT is also considered in order to improve the quality of life in patients with chronic liver diseases [7]. Hepatologists are guided by several scores to assess the indications for LT, in order to include the patients on a waiting list for LT. Currently, the MELD score (Model for End-stage Liver Disease) is considered the standard indicator of the liver severity status of the recipient and is used to predict short-term mortality in cirrhotic patients [8,9]. Patients with acute liver failure are eligible for urgent LT, in accordance with scoring systems like: King’s College criteria, and Clichy criteria [7].

Medical conditions that compose the list of indications for LT could be divided into three major categories according to the EASL guidelines: Acute liver failure (8%), chronic/end-stage liver diseases (cirrhosis and its complications: variceal hemorrhage, ascites, hepatorenal syndrome, and encephalopathy represent 57%), and primary liver malignancies (15%) [6].

Table 1 contains the main indications for LT, according to the underlying liver disease [6,7,8,9].

Even though medicine has recorded great progress in the transplantation field, and seen a rise in lives spared, there are still several situations which contraindicate LT. For example, the MELD scoring system was intensively studied in order to predict the benefit of LT. A MELD score of ≥15 represents a universally accepted value for listing a patient, since survival after the transplant is significantly higher [6,10]. In a multivariable analysis [11,12], a MELD score >25, combined with other factors (recipient older than 65 years, donor older than 50 years, male sex, retransplantation) are shown to be associated with poor survival. A MELD score between 15–17 is considered a transition point, while patients with a low MELD score (≤14) have a higher mortality rate within the first year after LT than patients on the waiting list, who have little or no progression [10].

Contraindications to LT are listed in Table 2 [7,9].

## 2. Cardiovascular Function before and after Liver Transplantation

### 2.1. General Considerations

According to the American Association for the Study of Liver Diseases (AASLD) [13], cardiovascular (CV) disease and renal failure are the most encountered causes of mortality and morbidity following LT. Van Wagner et al. showed in their study that CV disease is the major cause of short and long-term complications after LT, being responsible for 42% of deaths within 30 days following LT [14]. Moreover, several studies [15,16] have observed an incidence of approximately 11% for new CV events following LT over a period of 5 years, significantly higher than the general population. A meta-analysis of 12 studies [17] concluded that patients with LT have a 10-year risk of 13.6% of suffering from a CV event. As stated by the American Heart Association [18], cardiovascular disease is defined as ischemic heart disease, stroke, heart failure and thromboembolism.

### 2.2. Pathogenesis of CV Dysfunction

Patients with advanced liver disease are affected by subclinical CV dysfunction, reflected in systemic hemodynamic changes: high heart rate, increased cardiac output, and decreased systemic vascular resistance [19]. Cardiomyopathy associated with cirrhosis appears in the absence of other cardiac diseases and was defined in accordance with the international consensus committee at the World Congress of Gastroenterology in 2005, by the following criteria [20]:

- Systolic dysfunction: impaired contractility leads to increased ventricular filling pressure and low systemic vascular resistance, which become symptomatic under stress conditions like LT [21,22].
- Diastolic dysfunction: decreased relaxation leads to altered ventricular filling, and its severity has been proven to correlate with increased mortality following LT [21,22].
- Electrophysiological alterations (QT interval prolongation, inotropic and chronotropic incompetence, electrical and mechanical dissociation) are responsible for severe arrhythmias [22,23].

Moller et al. [22] sustain that approximately half of the cirrhotic patients have associated cardiomyopathy. Its presence may be responsible for other complications of cirrhosis (for example: hepatorenal syndrome, spontaneous bacterial peritonitis, acute heart failure after transjugular intrahepatic portosystemic shunt insertion) [24]. Cardiac dysfunction also contributes to the poor prognosis of patients after LT, taking into consideration the acute hemodynamic changes caused by reperfusion of the graft and the release of proinflammatory cytokines [25]. Furthermore, there is a well-known increased CV risk associated with chronic immunosuppressive treatment [23].

### 2.3. CV Assessment before LT

According to the European Society of Cardiology and Anesthesiology, LT is considered a non-cardiac surgery procedure with high risk for infarction and cardiac death in the first month postintervention [26]. Taking into consideration that donor availability is limited, it is mandatory to perform a thorough CV assessment before LT. Severe cardiac diseases represent a contraindication to performing LT.

The standard pre-LT work-up includes electrocardiogram and transthoracic echocardiography [6], in order to identify cardiac disorders that, untreated, would worsen the LT outcome. In addition, patients older than 50 years, or with cardiovascular risk factors (hyperlipidemia, hypertension, diabetes, and smoking), should undergo noninvasive stress testing and cardiologic evaluation, in order to assess underlying coronary artery disease [6,8,27]. Patients with proven ischemia from the stress test (for example: stress echocardiography with dobutamine) are referred to the multidisciplinary team for further investigations, like coronary angiography with revascularization, if there is more than 70% coronary artery obstruction [23]. So far, studies regarding stress echocardiography with dobutamine [28,29] are inconclusive, most of them stating that it is a weak predictor of major CV events after LT, and a normal result has a high negative predictive value (>90%) [30].

### 2.4. Factors Associated with CV Events after LT

Studies regarding screening for cardiac risk and predictors of CV events after LT are scarce. There are a lot of variables related to the recipient and the donor that influence the outcome of a patient with LT. Traditionally, due to its impact on long-term survival, CV disease was considered a relative of, or sometimes even an absolute contraindication for, LT [31].

A large multicenter cohort study including 32,810 patients with LT showed that major CV events at 30 and 90 days were independently predicted by age (more than 65 years), indication for LT (alcoholic cirrhosis, and non-alcoholic steatohepatitis), and preexisting CV and renal comorbidities (ischemic heart disease, myocardial infarction, heart failure, atrial fibrillation, and higher creatinine value) [32]. The MELD score was also higher among patients with early CV complications after LT [32]. These results were consistent with findings from other studies [14,30,33].

A recently published study [27] demonstrated that the prevalence of traditional CV risk factors among LT patients is around 23%; the most common being hypertension and diabetes. However, studies failed to demonstrate a significant association between the risk factors and mortality, due to CV events within the first 30 days after LT [14]. Several characteristics were associated with the risk of death long-term following LT: presence of coronary artery disease, with stenosis at coronarography and reduced ejection fraction (less than 50%), and portal vein thrombosis [27]. It has also been reported that the donor’s features influenced the perioperative mortality due to CV events: high donor body mass index, deceased vs. living donor, and cardiac death of the donor [14]. On the other hand, statistics have not found any correlation between sex, race, ethnicity, cerebrovascular comorbidities of the recipient, and mortality due to CV causes [14].

### 2.5. Early Perioperative CV Complications

LT constitutes major stress and affects preexisting CV dysfunctions, leading to different consequences [23]. Graft reperfusion is associated with acidosis, dyselectrolytemia, hypothermia, and hypotension [34]. A severe drop of systolic blood pressure can be seen in 30% of cases within the first five minutes and is defined as postreperfusion syndrome [25]. This hemodynamic instability is caused by the acute release of cytokines in the flush blood from the graft [25].

In a multicenter study published by VanWagner [14] regarding complications of LT, a 2.9% all-cause mortality within 30 days was encountered, and 42.1% of the deaths were from CV events. Approximately half of the deaths were caused by cardiac arrest, followed by stroke, heart failure and pulmonary embolism. These patients were older and associated with a higher MELD score, perioperative renal/respiratory failure, and pretransplant diabetes/hypertension. Acute coronary syndrome was observed during the first 10 days in 5.4% of the patients, in a study conducted by Nicolau-Raducu et al. [33]. A total of 86% of these patients were associated with more than two risk factors for CV disease before undergoing LT [33].

In addition, portal vein thrombosis induces endothelial dysfunction, and is associated with acute coronary syndrome [35]. Hypercoagulable state is a condition encountered after LT and might be the cause of myocardial infarction. Moreover, studies have described another complication in LT recipients, called dilated cardiomyopathy, which is a reversible condition associated with pulmonary edema [36].

### 2.6. Long-Term CV Risks

Studies concerning long-term complications are more rare than those focused on early morbidity. There are some factors that have to be taken into consideration. For example, unlike most CV complications that occur shortly after LT, due to impaired preexisting cardiovascular and hemodynamic status met in cirrhosis, the long-term risks are attributable to coronary artery disease [37]. The longer they live after LT, the greater the CV risk becomes for these patients, as well as for the general population. Studies have also revealed that survival at five years after LT was reduced in patients who experienced early postoperative CV complications [36,37,38]. Moreover, chronic immunosuppressive drugs are well-known risk factors for long-term morbidity due to atherosclerosis/coronary artery disease, dyslipidemia, hypertension, and diabetes mellitus type II [39]. Most regimens include calcineurin inhibitors (CNIs) like tacrolimus and cyclosporine, with cyclosporine having higher implications in metabolic and cardiovascular comorbidities than tacrolimus [40]. The mammalian target of rapamycin (mTOR) inhibitors like everlimus and sirolimus, have been approved as maintenance immunosuppression agents after LT. mTOR inhibitors may be given in combination with CNIs or conversion therapy in CNI-free regimens. The benefits of mTOR inhibitors in liver transplantation are related to renal function as they are considered renal sparing agents, while their efficacy is comparable to CNIs [41]. A cross-sectional study conducted by Nicolau-Raducu et al. [33], over a follow-up period of 3.4 years, demonstrated that CV morbidity during the first year was 15.2%, with a decrease to 3.9% for those patients who survived more than one year. It was noted that from a total of 389 patients, 59 patients had at least one cardiovascular event within the first year: heart failure, arrhythmia, cardiac arrest, or stroke. Late CV complications, following the first years, occurred in 14 cases: Five patients experienced myocardial infarction, with a mean time of 24 months after LT (these patients either had a coronary stent previously inserted or were associated with more than three risk factors); long-term complications also included: heart failure, atrial fibrillation, and peripheral vascular disease [33].

## 3. Metabolic Syndrome in Liver Transplantation

### 3.1. General Considerations

In order to improve survival after LT, the underlying metabolic and CV disorders should be diagnosed and treated before performing LT. Also, the new metabolic and CV diseases that appear after LT, in the early or late period, should be screened and treated. These complications have a higher prevalence in LT recipients in comparison with the general population [42,43].

Metabolic syndrome (MS) is defined by a combination of several disorders including hypertension, obesity, insulin resistance/diabetes mellitus (DM), and dyslipidemia. Its prevalence after LT is well documented in different studies and ranges between 44% [43] and 58% [44,45].

Regarding immunosuppression regimens, mTOR inhibitors lower the risk of metabolic syndrome compared to CNIs, having a positive effect on all components of the MS, with the exception of hyperlipidemia [41].

### 3.2. Factors Associated with Metabolic Syndrome after LT

Obesity prior to a transplant is considered a risk factor for metabolic syndrome after LT, and weight gain typically occurs within the first year after LT. Other risk factors which have been associated with post LT metabolic syndrome are: pretransplant diabetes, age, hepatitis C cirrhosis, cryptogenic cirrhosis, alcohol cirrhosis, hypertriglyceridemia, and low HDL cholesterol [45,46].

Since the metabolic syndrome develops early after the transplant, intervention should begin as soon as medically feasible [46].

### 3.3. Hypertension

Hypertension is not commonly encountered among patients with end-stage liver diseases, in whom the increase of cardiac output determines, on the contrary, a decrease of the systemic vascular resistance, along with low arterial pressure. Surprisingly, arterial hypertension has been described in LT recipients, with a prevalence reported in the literature between 40% [47] and 85% [15]. Rabkin et al. noticed that half of the patients with LT have systolic blood pressure >140 mmHg and/or a diastolic blood pressure >90 mmHg in the first six months [40]. The mechanisms are multifactorial and mainly related to immunosuppressants, with hypertension being more frequent in patients taking cyclosporin than tacrolimus [42]. Therefore, if hypertension develops, switching treatment with cyclosporin to tacrolimus may be considered [48]. An early diagnosis of hypertension, lifestyle changes, and antihypertensive medication (with calcium antagonists as the first choice, as they do not impact hepatic metabolism of immunosuppressants) are essential measures that increase long-term survival of LT patients [49].

### 3.4. Obesity

Overweight and obesity are defined by the World Health Organization as a body mass index (BMI) higher than 25 kg/m^2^ and 30 kg/m^2^, respectively. There are two possible situations that can impact the survival of LT recipients: patients are either overweight/obese before undergoing LT, or they gain weight after LT. According to studies, up to 30% of the patients waiting for LT have a BMI >30 kg/m^2^ and associate with other risk factors like diabetes, high serum creatinine, and dyslipidemia [44]. Obese patients continue to have a high BMI even after three years after LT [50]. Morbidity and mortality at five years are significantly higher among obese patients compared to non-obese patients (mainly due to CV events) [51]. Obesity after LT has been reported to range between 21% [52] and 43% [43], with the highest incidences during the first year. Patients gain weight after LT because their catabolic state induced by cirrhosis is no longer present and therefore their appetite increases. Another explication for weight gain is the role of immunosuppression. It is well-known that corticosteroids induce overeating. Cyclosporine is associated with a higher weight gain than tacrolimus within the first year; afterwards, there is no difference between them regarding BMI [42]. Age is considered a risk factor, since patients older than 50 years tend to gain more weight in the first two years than younger patients [50].

### 3.5. Insulin Resistance/Diabetes Mellitus (DM)

All solid organ transplantations are associated with hyperglycemia and new-onset DM (newly diagnosed after the transplant) [53]. Studies have discovered that almost 80% of the patients with cirrhosis have impaired tolerance to glucose and 20% of them are diagnosed with diabetes [43]. The new-onset DM is defined according to the World Health Organization as: symptoms of diabetes (polydipsia, polyuria, and weight loss) plus random plasma glucose ≥200 mg/dL, or fasting plasma glucose ≥126 mg/dL, or 2-h plasma glucose after a 75 g oral glucose intake ≥200 mg/dL, or HbA1c ≥6.5% [53,54]. The factors incriminated in the appearance of DM diagnosed after transplantation are immunosuppressive regimens (calcineurin inhibitor and steroids, depending on dose), infections, rejection requiring high-doses of immunosuppressants, and surgery-associated stress. Tacrolimus has a higher risk of inducing new-onset DM than cyclosporine [38]. In LT recipients, particularly newly diagnosed, DM was more frequently seen in patients being overweight before LT, or that had virus C infection or non-alcoholic fatty liver disease [55,56].

Patients need insulin treatment before undergoing LT and in the immediate after LT period. Hyperglycemia may be transient after transplantation, but sustained hyperglycemia (more than six months after LT) is reported to increase morbidity and mortality over a long-term period [57].

### 3.6. Hyperlipidemia

Due to impaired liver function, lipid levels are low in most patients before LT. Dyslipidemia may be seen in only 8% of the patients who have cholestatic disease and in whom liver synthesis function is still preserved [38,43].

Gisbert et al. reported a 66% incidence of hyperlipidemia after LT [58]. Hypertriglyceridemia is more commonly seen in LT recipients compared to hypercholesterolemia (19% vs. 59%) [43,59]. Hypertriglyceridemia may be encountered mostly isolated, rarely associated with hypercholesterolemia, and usually appears within one month after LT, remaining unchanged over the first year [58]. Lipid disorders have multifactorial causes, including increased BMI, lifestyle, impaired glucose tolerance and insulin resistance, genetic predisposition, and reduced kidney function [60]. Lipid metabolism is modified by immunosuppressants, which independently increase cholesterol and triglycerides, this effect being dose-dependent. Studies have shown that cyclosporine has a more profound effect than tacrolimus and that switching regimens may improve dyslipidemia [61]. High serum LDL-cholesterol levels are additional risk factors for atherosclerotic disease in LT recipients [60].

## 4. Conclusions

Cardiovascular complications and metabolic alterations are responsible for substantial morbidity and mortality during early and long-term follow-up among LT recipients. Therefore, it is crucial to identify and treat underlying CV dysfunction prior to transplantation.

As the demand for liver transplantation is overwhelming, we consider that the selection of patients on the wait-list for LT must be thoroughly made in order to improve outcomes after LT.

Corroborating all the information from the literature, we conclude that management of patients with LT should also focus on lifestyle modification, decrease of BMI and immunosuppressive strategies to lower rates of diabetes, and hypertension and hyperlipidemia after transplant, especially in persons with multiple risk factors present prior to the transplant.

## Figures and Tables

**Table 1:** Indications for liver transplantation.

Acute Liver Failure	Hepatitis A/B
	Intoxication (e.g., acetaminophen)
	Wilson’s Disease
	Budd–Chiari Syndrome
Chronic liver failure: Non-cholestatic cirrhosis	Hepatitis B/C
	Autoimmune hepatitis
	Alcohol-induced cirrhosis
Chronic liver failure: Cholestatic cirrhosis	Primary biliary cirrhosis
	Primary sclerosing cholangitis
Chronic liver failure: Metabolic	Wilson’s disease
	Hemochromatosis
	α-1 antitrypsin deficiency
	Amyloidosis
	Cystic fibrosis
	Tyrosinemia
Chronic liver failure: Vascular	Budd–Chiari syndrome
Malignant disease	Hepatocellular carcinoma (within Milan criteria)
	Fibrolamellar carcinoma
	Epithelioid hemangioendothelioma
	Cholangiocellular adenocarcinoma
	Neuroendocrine liver metastases
Benign liver tumors	Adenomatosis
Liver transplantation in pediatric patients	Biliary atresia
	Byler’s disease
	Alagille’s syndrome
	Neonatal hepatitis/neonatal viral hepatitis
	Autoimmune hepatitis
	Hepatoblastoma
Other indications	Primary oxalosis
	Glycogen storage diseases
	Hyperlipidemia
	Polycystic liver disease

**Table 2:** Contraindications for liver transplantation.

Absolute Contraindications	Relative Contraindications
Severe cardiopulmonary diseases	Cholangiocarcinoma
Uncontrolled extrahepatic malignancy	Advanced age (>65)
Active alcohol/substance abuse	Severe obesity/malnutrition
Acute alcoholic hepatitis	Diffuse portal vein thrombosis
Uncontrolled sepsis	
Lack of psychosocial support/inability to comply with medical treatment	
Brain death	
